# North American and Andean Indigenous Plant-Based Foods and Their Contemporary Scientific Rationale for Health Improvement

**DOI:** 10.1016/j.cdnut.2025.107597

**Published:** 2025-11-15

**Authors:** Lena Gálvez Ranilla, Kalidas Shetty, Mercedes Morin

**Affiliations:** 1 Laboratory of Research in Food Science, Universidad Catolica de Santa Maria, Arequipa, Peru; 2 Escuela Profesional de Ingeniería de Industria Alimentaria, Departamento de Ciencias e Ingenierías Biológicas y Químicas, Facultad de Ciencias e Ingenierías Biológicas y Químicas, Universidad Catolica de Santa Maria, Arequipa, Peru; 3 Department of Microbiological Sciences, North Dakota State University Fargo, Fargo, ND, United States; 4 Environmental and Conservation Sciences Program, North Dakota State University Fargo, Fargo, ND, United States

**Keywords:** traditional food systems, Indigenous plant foods, bioactive compounds, health, North America, Andean grains

## Abstract

Plant-based food systems have been an integral part of human ecology since the dawn of domestication across all continents from over 12,000 y ago. This served the needs of human health and wellness from whole plant form to the use of parts in a range of water-based extracts to dry form or fermentation-based applications. This conference paper focuses on some examples of plant-based foods in the Americas that have been used since ancient times among indigenous North American and Andean societies. In contemporary times, different factors have decrease in their consumption, leading to several diet-related health impairments such as noncommunicable chronic diseases and nutritional deficiencies. This challenge is also compromising the preservation of indigenous food diversity which also includes all the indigenous concepts associated with traditional food systems from both regions of the Americas. The objective of the current paper is to summarize the scientific advances building from the conference presentation regarding the nutritional and health-relevant potential of some indigenous foods from these regions to contemporary health challenges. Some indigenous food concepts and examples from North America such as the Three Sisters crops, wild rice, and some native berries, and diverse Andean grains have been discussed. In addition, some initiatives aimed at preserving indigenous food diversity and associated food systems in each region have been integrated. The scientific evidence presented here is increasingly validating the knowledge of indigenous people from across Americas linked to the consumption of native foods for maintaining good nutrition and health, which in turn would reconnect indigenous communities with their culture and traditions. More scientific research and efforts from multiple stakeholders, and primarily involving indigenous communities, are needed to continue the revitalization of indigenous food systems.

## Introduction

Plant-based food systems have been an integral part of human ecology since the onset of agriculture ∼12,000 y BC [1]. Diets among Neolithic Eurasian populations were high in starchy cereals, supplemented by vegetables and domestic animals [1]. Both cultivated and gathered plants provided around 75% of the total consumed calories [2]. In case of the Americas, agriculture developed independently in both North and South America within a shorter time period in comparison with Europe [3]. In fact, Eastern North America and Andean regions have been recognized as independent centers of plant food domestication worldwide [[4], [5], [6]]. Several environmental and geographical conditions may have played a role in dietary patterns among early civilizations in these regions, especially before the European colonization.

Seed plant species of squash (*Cucurbita pepo* ssp. *ovifera*), sumpweed (*Iva annua*), sunflower (*Helianthus annuus*), and chenopod (*Chenopodium berlandieri*) were first domesticated in Eastern North America during a period of 2000–1000 BC. [7]. These plants along with other seed species were then considered staple food crops among local settlements from 250 BC to 200 AD [7]. Later, a shift to maize-centered agriculture occurred between 800 and 1100 AD [7]. In contrast, the forests with variable conifer species from Northwestern North America region, provided diverse plant and animal-based foods to indigenous people over millennia [8]. Several plant species, such as fruits, seeds, greens, nuts, roots, among others, have been documented to be used by indigenous groups from this region [9,10].

The Andean region is characterized by the presence of the Andes, which is the longest cordillera in the world, encompassing 7 countries in South America (Venezuela, Colombia, Ecuador, Peru, Bolivia, Argentina, and Chile) [11]. This region shows extreme geographical heterogeneity, with unique ecosystems which have fostered adaptations of agriculture linked to dietary changes among Andean people over the past 7000 y [12,13]. For example, southern Andean people from 3400 y ago made agriculture possible at elevations around 3800 m using the thermoregulatory effects of Titicaca Lake Andean basin [14,15]. Human diets in this region were mainly composed of C3 plants such as quinoa (*Chenopodium quinoa*), tubers (*Solanum tuberosum*, *Oxalis tuberosa*) and domestic camelids (*Lama glama* L.) [15]. Pre-Hispanic indigenous communities built complex terracing and irrigation systems through coordinated communal labor, which allowed the intensive production of several crops at highland conditions [16]. These structures were later expanded to new areas during the Inca empire (1430 AD), and agriculture reached maximum development [17]. Maize acquired a high importance for food security and religious needs [18]. In the Inca period, additional sociodemographic factors were also determinant on food production [12,16].

In addition to the advance of agriculture, people from both North American and Andean regions developed and shared a similar holistic perception or “cosmovision” about their connection with nature [8,19]. This ancient approach led to the origin of unique “traditional food systems” in both geographical areas. This system is defined as all foods available from natural resources that are culturally significant to a specific cultural group which includes cultivation practices, harvesting, processing techniques, uses, associated teachings/knowledge, and nutritional aspects [20]. Indigenous knowledge emphasizes the interconnectedness of all living beings with nature, and that humans should be guided by and have respect toward Mother Earth [21]. This is an idea that has been labeled by Western societies as kincentric ecology, which underscores the symbiotic relationship between indigenous communities and their environments, fostering sustainable practices that preserve biodiversity [22]. Accordingly, foods not only had a nutritional role, but also showed cultural meaning among American indigenous communities. This was expressed in several practices and rituals such as the sustainable design of estuarine root gardens by North American indigenous groups, or the “payment to Pachamama” (Mother Earth in the “Quechua” native language) as a sign of gratitude to earth before harvesting plant foods in Andean localities [8,23].

European colonization disrupted traditional food systems in both North American and Andean indigenous groups, negatively impacting their food security and health. Most native crops were banned or replaced, and people were dispossessed of lands or subjected to forced resettlements [8,24,25]. Currently, factors such as sociocultural changes and the economic model are aggravating the disconnection of indigenous people with their traditional food systems, which in turn is affecting their health [26]. North American indigenous communities are experiencing substantial health disparities, food insecurity, and elevated poverty rates [27]. The undernutrition of this group before the 1970s has been replaced by overnutrition from the current Western diets, which are low in micronutrients, and high in refined calorie-dense macronutrients [28]. Accordingly, this group has shown heightened susceptibility to noncommunicable chronic disease (NCDs). American (Indian/Alaskan) indigenous adults are nearly 3 times more likely to be diagnosed with diabetes in comparison to their non-Hispanic white counterparts [29]. A study conducted by the Centers for Disease Control and Prevention revealed that 16% of Indigenous North American (Indian/Alaskan) individuals aged 18 and above are diagnosed with diabetes, a staggering contrast to the 8.5% prevalence among non-Hispanic white adults [30].

The total deaths due to NCDs among Andean people have increased from around 64%–83% (2010) to 72%–85% (2019) [31]. Cardiovascular diseases (CVD) and cancer are the main causes of death, whereas major risk factors are linked to diet-related health problems such as hypertension, obesity, and hyperglycemia [31]. Concurrently, there is a double nutritional burden in relation to the deficiency of important micronutrients such as iron, particularly among vulnerable populations such as children and pregnant women [32]. The current dietary patterns among Andean people have changed, showing preference for ultraprocessed foods [33]. The purchase per capita of ultraprocessed foods between 2000 and 2013 has significantly increased in Andean countries exhibiting a positive correlation with the incidence of obesity and the increase of metabolic diseases such as diabetes [33].

Despite the growing awareness and efforts related to these health challenges, there remains a critical need for research that not only targets these underlying health disparities but also uses indigenous knowledge to address these challenges. Indigenous traditional food systems contain significant and extensive knowledge of local ecology that has been underutilized in contemporary society. This is an emerging approach that some authors are defining as “indigenous cultural health” which considers critical elements such as “country” (lands and ecosystems), “people,” and “culture” (knowledges, identity, language) in an integrated and holistic way to promote health initiatives among indigenous communities [34]. The combination of these elements integrated with modern scientific research knowledge may have a significant influence on the health of the community, including physical, emotional, mental, and spiritual health [35].

**FIGURE 1:**
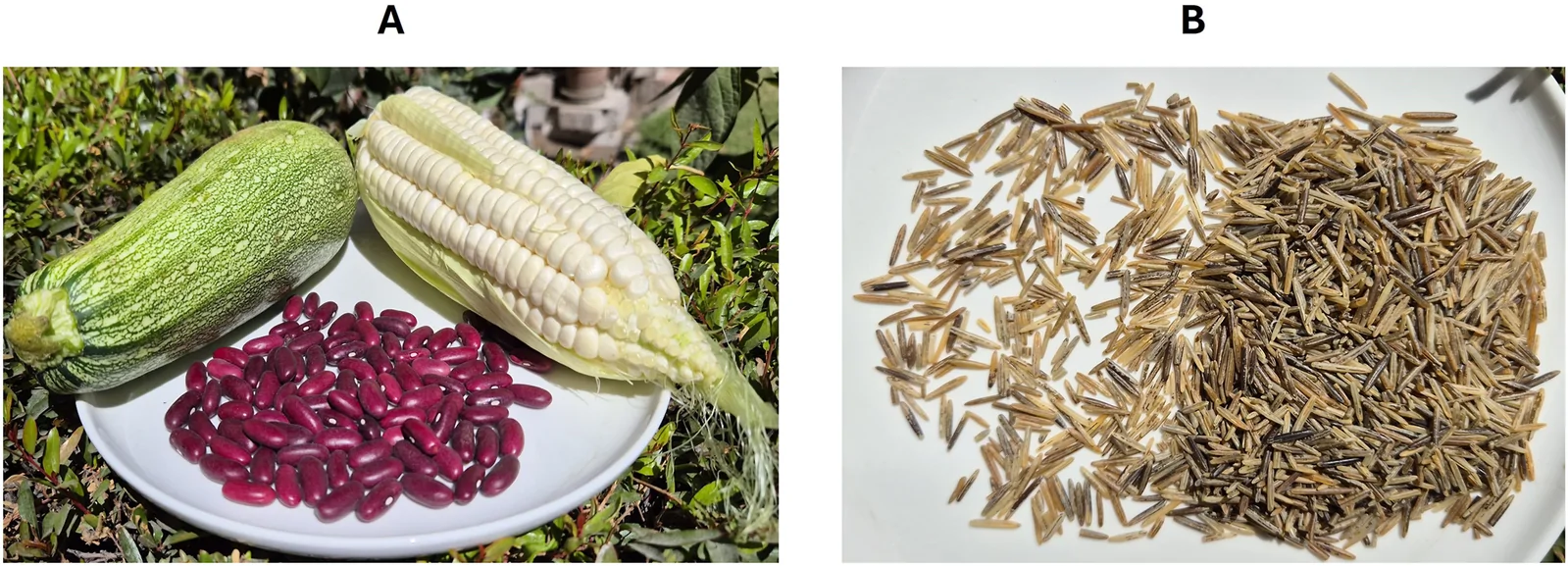
(A) Three Sisters crops (Squash, *Cucurbita* spp.; Beans, *Phaseolus vulgaris*; Maize, *Zea mays* L.). (B) Wild rice (*Zizania* spp.).

**FIGURE 2:**
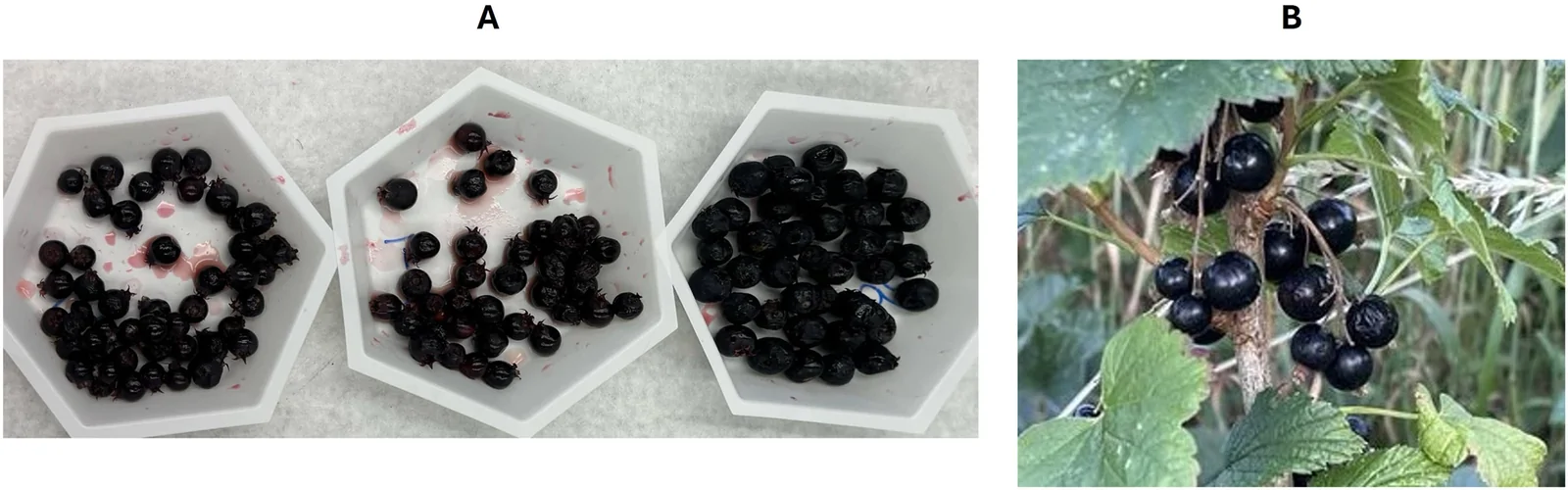
Pictures of some North American berries: (A) Saskatoon berries (*Amelanchier alnifolia* Nutt.). (B) Black currants (*Ribes nigrum* L.).

**FIGURE 3:**
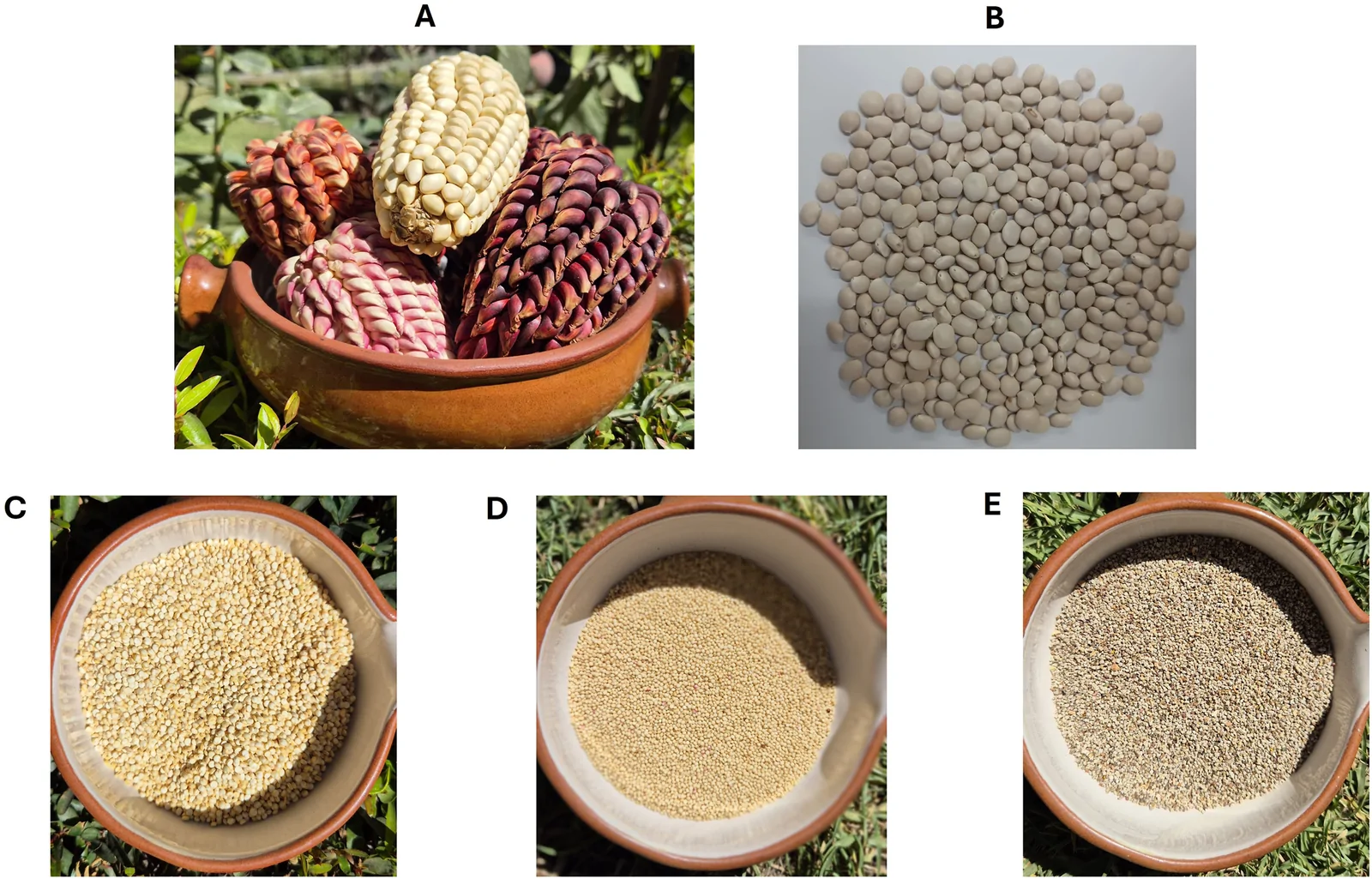
(A) Andean maize (*Zea mays* L.). (B) Andean lupin or *tarwi* (*Lupinus mutabilis* Sweet). (C) Quinoa (*Chenopodium quinoa* Willd.). (D) Amaranth (*Amaranthus caudatus* L.). (E) *Cañiwa* (*Chenopodium pallidicaule* Aellen).

The main objective of the current paper is to summarize the presentation of the 2023 Native American Nutrition conference and highlight the scientific evidence about the nutritional potential and health-relevant functionality of some examples of indigenous foods from North American and Andean regions. For this purpose, the Three Sisters system (Figure 1A), wild rice (Figure 1B), and native berries (Figure 2), along with the case of important Andean grains (Figure 3) were selected as food examples from both regions of the Americas. In addition, some strategies aimed at revitalizing traditional North American and Andean food systems for health purposes have been discussed.

## Nutritional Composition and Health-Relevant Functionality of Some North American Indigenous Foods

### Nutritional composition

The nutritional composition of some North American indigenous foods including the Three Sisters crops (maize, beans, and squash), wild rice, and some native berries is shown in Table 1. The Three Sisters crop system is an intercropping traditional agricultural practice developed by native tribes since pre-Hispanic times [36]. It consists of planting diverse varieties of maize (*Zea mays* L.), beans (*Phaseolus vulgaris*), and *Cucurbitaceae* species such as squash and pumpkin in a polyculture system [36]. This cultivation method was originally developed in Mesoamerica where it is known as “milpa” and then later spread to the rest of North America where it was likely adopted in 1070 AD according to archeological information [[37], [38], [39]]. Foods included in the Three Sisters system show proper macro and micronutrient balances [40]. Beans stand out for their high protein (26.8%, dried weight, DW), dietary fiber, minerals and vitamins levels, whereas maize is a source of lipids (mostly polyunsaturated forms), carbohydrates, and dietary fiber (Table 1) [41]. Pumpkin (flesh) is remarkable due to its high contents of calcium, phosphorus, potassium, vitamins A, B, C, and E, along with protein and dietary fiber (Table 1).

**TABLE 1:** Nutritional composition of some North American indigenous foods and berries (in 100 g of whole raw edible portion, dry weight, DW)

Compound	Unit	Maize (yellow) (*Zea mays* L.)	Kidney beans (*Phaseolus vulgaris*)	Pumpkin (*Cucurbita spp.)*	Wild rice (*Zizania acuatica*)	Saskatoon (*Amelanchier alnifolia* Nutt.)	Chokecherry (*Prunus virginiana*)	Red currants (*Ribes* spp.)	American cranberry (*Vaccinium macrocarpon*)
Origin		NR	NR	NR	Canada and United States	“Smoky” cultivar- Canada	Canada^1^	Poland^2^	Latvia
Macronutrients									
Protein	g	10.5	26.8	11.9	14.5	6.5	6.6	8.9	NR
Total lipids	g	5.3	0.9	1.2	0.9	2.4	0.6	2.1	NR
Total carbohydrates	g	82.9	68.0	77.4	81.8	90.7	90.1	85	NR
Total dietary fiber	g	8.1	28.2	6.0	6.8	29.1	26.8	27.9	NR
Ash	g	1.3	4.3	9.5	1.5	3.1	2.7	4.0	1.6
Micronutrients									
Calcium, Ca	mg	7.8	162	250	25	206	181	214	75
Iron, Fe	mg	3.0	9.3	10	1.7	4.7	2.1	6.5	5.3
Magnesium, Mg	mg	142	159	143	127	120	81	84	49
Phosphorus, P	mg	234	461	524	374	99	202	286	63
Potassium, K	mg	320	1599	4048	266	795	1142	1786	533
Sodium, Na	mg	39	27	12	6.4	2.4	15	6.5	NR
Zinc, Zn	mg	2.5	3.2	3.8	2.9	0.8	1.0	1.5	1.2
Copper, Cu	mg	0.4	1.1	1.5	0.4	NR	0.6	0.7	0.4
Vitamin C, total ascorbic acid	mg	0.0	5.1	107	NR	17	16.6	266	146
Complex B^3^	mg	5.9	5.0	14	0.7^4^	19	4.3	2.2	NR
Vitamin A, RAE	μg	12	0.0	5071	NR	53	24	13	NR
Vitamin E (α-tocopherol)	mg	0.5	0.2	13	0.2	5.5	1.1	0.6	NR
References		[41]	[41]	[41]	[42,43]	[44]	[41,45]	[41,46]	[47,48]

Abbreviations: NR, not reported; RAE, Retinol Activity Equivalent.

1 Macronutrients data from Canadian sample.

2 Macronutrients data from Poland sample.

3 Includes the sum of thiamin, riboflavin, niacin, pantothenic acid, Vit B-6-piridoxin, total folate (B9).

4 Only thiamin and riboflavin.

Wild rice (*Zizania* spp.) is an ancient cereal that has been a staple food for many indigenous American tribes throughout the Great Lakes region [49]. Besides its nutritional potential, it holds great significance at spiritual and cultural levels among indigenous communities [49]. According to Table 1, the protein contents of *Z. acuatica* (14.5% dried weight, DW) are higher than those found in maize and pumpkin (10.5%–11.9% DW), whereas the dietary fiber and mineral contents are almost comparable to levels observed in maize. Compared with white rice (*Oryza* spp.), wild rice has shown almost 5 and 2 times higher dietary fiber and protein contents, respectively [43].

Native berries have been used for centuries for both food and medicinal purposes among North American indigenous people [50]. As foods, berries were mostly consumed fresh, dried, or incorporated into different traditional preparations [50]. Saskatoon berries (*Amelanchier alnifolia* Nutt.) are fruits native to the Northwest territories, the southern Yukon, the Canadian Prairie Provinces, and the northern plains of the United States [51]. Chokecherry (*Prunus virginiana*) is another berry often used by indigenous American tribes before the colonization such as the Blackfoot [52]. Several *Ribes* spp. types, mostly wild species, have also been reported in North American area [51]. *Vaccinum macrocarpon*, known as large cranberry, corresponds to the American cranberry species [53]. Nutritionally, above berries are rich sources of dietary fiber (26.8%–29.1% DW), minerals such as potassium (533–1786 mg/100 g DW), and vitamin C (16.6–266 mg/100 g DW). Red currants exhibit the highest vitamin C contents, whereas saskatoon berries show comparable vitamin B levels to those of pumpkin (Table 1). Carbohydrate concentrations are also high (85%–90.7% DW) (Table 1), and are mainly composed of digestible sugars such as glucose and fructose [54]. However, the overall composition of berries makes them low in calories, showing potential as functional foods [54].

### Health-relevant functionality

Table 2 summarizes information about the bioactive composition and health-relevant properties reported in the Three Sisters crops and wild rice. Maize and beans contain different bioactive compounds including polyphenols, carotenoids, tocopherols, phytosterols, dietary fiber, and oligosaccharides [[55], [56], [57], [58], [59],^60^,^61^] (Table 2). Saponins, phytates, and lectins have also been reported in bean kernels [62]. In the case of maize, the purple-pigmented type has received more attention. Several in vitro and in vivo studies highlight the antioxidant, antimutagenic, antihyperglycemic, and antihyperlipidemic activities of maize, which have been mainly correlated with its anthocyanin contents [63,64,65]. However, other maize compounds such as bran hydroxycinnamic acid amides (HCAAs) and feruloylated oligosaccharides exhibited antihyperglycemic and anti-inflammatory effects [66,67]. The intake of differently pigmented beans has shown benefits against obesity, diabetes, and cardiovascular disease risk according to in vivo studies and clinical trials [68,[69], [70], [71]]. Moreover, specific bean fractions such as phenolic-rich extracts, isolated peptides, and lectin have been associated with antihyperglycemic, antihypertensive, and antitumor activities, respectively [72,73,74].

**TABLE 2:** Bioactive compounds and reported health-relevant properties associated to some North American indigenous foods

Food	Bioactive compounds	Reported bioactivity		
		In vitro	In vivo	Studies in humans
Maize (*Zea mays* L.)	- Oligosaccharides [55] - Phenolic and derivative compounds: hydroxycinnamic acid amides (HCAAs) [56]; flavonoids (anthocyanins, flavonols, flavones C-glycosides, flavanol-anthocyanins) [57,58]; hydroxybenzoic acid derivatives [58]; phlobaphenes [59] - Carotenoids (lutein, zeaxanthin, β-cryptoxanthin, β-carotene) [58] - Bioactive peptides, resistant starch, tocopherols, phytosterols [59]	- Antimutagenic activity correlated with high-anthocyanin blue maize extracts [63] - Antioxidant and antimelanogenic activities linked to maize bran HCAAs [75] - Antihyperglycemic potential of maize HCAAs and aqueous extracts (inhibition of α-glucosidase) [66,76]	- Anti-inflammatory effect of maize bran feruloylated oligosaccharides by inhibiting inflammation caused by influenza (mice model) [67] - Reduction of oxidative stress at liver level linked to corn anthocyanins (rat model) [64] - Control of hyperglycemia and hyperlipidemia of high anthocyanin purple corn extract (mice model) [65]	- Positive adjuvant effect of purple maize extract (rich in antioxidants) for Crohn’s disease remission under a monoclonal antibody’s treatment (patients with inflammatory bowel disease) [77]
Bean (*Phaseolus vulgaris*)	- Phenolic compounds: phenolic acids (hydroxycinnamic acids, hydroxybenzoic acids), flavonoids (flavanols, flavanones, anthocyanins), proanthocyanidins [60] - Phytosterols, dietary fiber, tocopherols [61] - Saponins, phytates, oligosaccharides, lectins [62] - Carotenoids, ascorbic acid [78]	- Antioxidant activity linked to phenolic compounds, carotenoids, and ascorbic acid [78] - Antihyperglycemic and anti-inflammatory effects (seed coat phenolic-enriched extracts) [72] - Cancer cell proliferation inhibitory effect correlated with bean phenolic contents [79]	- Obesity prevention, regulation of glucose-insulin homeostasis, and anti-inflammatory effects linked to white bean intake (rat model) [68] - Antihypertensive and natriuretic effect of a bean peptide fraction (rat model) [73] - Antitumor activity of phytohemagglutinin (lectin) purified from red kidney bean (mice model) [74]	- Weight loss in individuals with obesity under regular intake of white bean extract [69] - Attenuation of postprandial insulin and improvement of antioxidant status in adults with metabolic syndrome after intake of meal with black bean (partially related with the dietary fiber and antioxidant contents) [70] - Antidiabetic potential, decrease of cardiometabolic risk, and prebiotic effects in healthy adults after evening intake of brown beans [71]
Squash (*Cucurbita* spp.)	- Flesh (phenolic compounds, D-chiro-inositol, myo-inositol, protein-bound polysaccharides, carotenoids, cucurbitacin glycosides C, E); seeds (proteins, phenolic compounds: lignans, isoflavones; β-sitosterol; tocopherols); peel (pectin, dietary fiber) in *C. pepo* [[80], [81], [82], [83]] - Flowers (carotenoids, phenolic compounds) in *C. maxima, C. moschata*, and *C. pepo* [84]	- Antioxidant activity in peels of *C. maxima*, *C. pepo*, *C. moschata*, *C. ficifolia*, and *C. argyrosperma* [83] - Cardiovascular disease prevention through antiplatelet activity of yellow *C. pepo* extract [85] - Antioxidant, antihyperglycemic (α-glucosidase and α-amylase inhibition), and antihypertensive (ACE-I inhibition) effects (flesh) [76]	- Antihyperglycemic effect via modulation of gut–liver axis and correlated with soluble dietary fiber of pumpkin flour (mice model) [86] - Dyslipidemia modulation through the hypotriglyceridemic and hepatoprotective effect of *C. moschata* seed flour (hamster model) [87] - Benign prostatic hyperplasia prevention of phytosterols isolated from *C. pepo* seed oil (rat model) [88]	- Oxidative stress protection due to consumption of fresh *C. ficifolia* (flesh) in patients with diabetes [89] - Hypoglycemic effect in patients with diabetes after intake of *C. maxima* flesh powder [90] - Decrease of risk of cardiovascular diseases via improvement of HDL cholesterol in postmenopausal women after intake of *C. pepo* seed oil supplement [91]
Wild rice (*Zizania* spp.)	- Phenolic compounds (phenolic acids; flavonoids: anthocyanins, flavanols, flavones, flavanones, isoflavones; proanthocyanidins) [92] - Phytosterols, γ-oryzanol, γ-aminobutyric acid [93] - Tricin (methylated flavone) in leaf blades of *Z. latifolia* [94]	- Antioxidant activity correlated with the proanthocyanidin fraction of *Z. latifolia* [95] - Anti-inflammatory, antioxidant, antiallergic, and skin-whitening effects of ethanolic extracts of *Z. latifolia* [96]	- Protection against nonalcoholic fatty liver disease of tricin-enriched *Z. latifolia* extract by reduction of oxidative stress, inflammation, and lipid disorders in liver (mice model) [97] - Improvement of insulin resistance, antihyperlipidemic and anti-inflammatory effects of *Z. latifolia* seeds (rat model) [98] - Antiatherogenic and cholesterol-lowering effects of *Z. palustris* diet (mice model) [99]	- Not found

Unless indicated, all information corresponds to the edible food fraction.

Abbreviations: ACE-I, angiotensin I-converting enzyme.

Bioactive compounds in species from the *Cucurbita* genus are not only distributed in the flesh but also are found in other edible plant parts such as seeds, peel, and flowers (Table 2). *C. pepo* flesh has polyphenols, carotenoids, and cucurbitacin glycosides (triterpenoids), whereas seeds are rich in polyphenols, phytosterols, tocopherols, and peel contains dietary fiber [[80], [81], [82], [83]]. Carotenoids and phenolic compounds have been found in *C. maxima*, *C. moschata*, and *C. pepo* flowers [84] (Table 2). In vitro studies in cooked extracts from various pumpkin types (flesh) have revealed their potential for the management of hypertension and hyperglycemia through the inhibition of the angiotensin I-converting, α-amylase, and α-glucosidase enzymes [76]. The antioxidant and hypoglycemic potential were later confirmed in patients with diabetes after the consumption of *C. ficifolia* and *C. maxima* flesh [89,90]. Other squash compounds, such as phytosterols derived from seed oil, have been related to the prevention of benign prostatic hyperplasia [88]. In addition, the intake of a seed oil supplement from *C. pepo* decreased the risk of CVDs through the increase of HDL cholesterol levels in postmenopausal women [91].

Wild rice has several bioactive substances including diverse polyphenols, and lipophilic compounds such as phytosterols and γ-oryzanol [92,93] (Table 2). In addition, wild rice has shown 10–15 times higher antioxidant activity and higher phytosterol levels than white rice [43]. A methylated flavone derivative named tricin has been identified in leaf blades of *Z. latifolia*, and has shown in vivo anti-inflammatory, antioxidant effect, and fatty liver disease protection [94,97]. The long-term intake (24 wk) of a wild rice (*Zizania palustris* L.) diet (as carbohydrate source) by LDL-receptor-deficient mice reduced the amounts of plasma cholesterol, the LDL, and the VLDL in comparison with the control treatment (with sucrose and corn starch as carbohydrate sources) [99]. Despite these advances, studies have mainly focused on the Chinese species (*Z. latifolia*) rather than in North American species such as *Z. aquatica*, *Z. palustris*, and *Z. texana* [100].

North American indigenous traditional medicine has used berry fruits or other plant parts for treating or preventing various ailments. For example, saskatoon (*A. alnifolia* Nutt.) berries were used to disinfect and prevent miscarriages [50]; chokecherry (*Prunus virginiana*) roots, barks, or fruits were consumed as tea and used to treat tuberculosis or stomach pain [101,102]; head colds were treated by smoking ground chokecherry bark like tobacco [102]. The scientific research is increasingly validating this indigenous knowledge, and some advances are shown in Table 3. Berries' main bioactive compounds correspond to the phenolic fraction which has been associated with different biological activities [46,50,103,104] (Table 3). The intake of saskatoon berry power rich in anthocyanins has shown in vivo anti-inflammatory and antidiabetic effects along with potential for gut microbiome modulation [105]. Polyphenols from chokecherry and currants (*Ribes* spp.) were correlated with their in vitro antioxidant activity [46,106]. Cranberry (*V. macrocapon*) phenolic compounds showed antimicrobial effect against *Escherichia coli* by inhibiting cell adhesion [107].

**TABLE 3:** Bioactive compounds and reported health-relevant properties associated to some North American indigenous berries

Indigenous food	Bioactive compounds	Reported bioactivity		
		In vitro	In vivo	Studies in humans
Saskatoon (*Amelanchier alnifolia* Nutt.)	- Phenolic compounds: flavonoids (anthocyanins-cyanidin derivatives, flavonols, flavanes-proanthocyanins), phenolic acids (hydroxycinnamic acids) [103,108] - Chlorophylls, tocopherols. triterpenoids, carotenoids [51]	- Antioxidant activity correlated with phenolic, triterpenoid, and carotenoid compounds [109] - Anti-inflammatory effect and reduction of diabetic microvascular complications linked to *A. alnifolia* nonpolar fraction [50] - Improvement of glucose uptake via an insulin-like effect linked to *A. alnifolia* polar fraction [50]	- Gut microbiome modulation, anti-inflammatory and antidiabetic effects of Saskatoon berry powder likely linked to anthocyanins and derived metabolite protocatechuic acid (mice model) [105] - Reduction of gut dysbiosis, diabetic, and inflammatory markers in a dose-dependent manner by the intake of Saskatoon berry powder (insulin-resistant mice model) [110] - Metabolic syndrome improvement likely linked to cyanidin glycosides (rat model) [111]	- Not found
Chokecherry (*Prunus virginiana*)	- Phenolic compounds (phenolic acids, anthocyanins, proanthocyanidins, flavanols) [50] - Carotenoids [50]	- Antimicrobial effect (bark extract) against methicillin-resistant *Staphylococcus aureus* (MRSA) under wound infection synthetic conditions [112] - Anti-inflammatory effect and reduction of diabetic microvascular complications linked to chokecherry polar and nonpolar fractions, respectively [50] - Antioxidant effect correlated with the total phenolic content [106]	- Not found	- Not found
Currants (*Ribes* spp.)	- Phenolic compounds (phenolic acids, flavanones, flavonols, flavones) in white, red and black currants [46] - Diterpenoids (carnosol) in white, red, and black currants [46] - Sarmentosin (nitrile glycoside) and derived hydroxycinnamoyl esters [113]	- Antioxidant activity correlated with the catechin, sinapic acid, rutin, and hesperidin contents in white, red, and black currants [46] - Higher lipid peroxidation inhibition in *R. nigrum* leaves than fruit extracts likely correlated with phenolic acids and flavonoids found in leaves [114] - Antihyperglycemic (α-glucosidase and α-amylase inhibition) and antihypertensive (ACE-I inhibition) activities of *R. rubrum* aqueous extracts [115]	- Neuroprotective, antioxidant, and anti-inflammatory effects in synergy with donepezil for memory deficit prevention. Aqueous *R. nigrum* extract by gavage administration (mice model) [116] - Anti-inflammatory, antioxidant, and antiplatelet effects of *R. rubrum* ethanolic extracts (diabetic rat model) [117] - Modulation of obesity-linked inflammation after intake of *R. rubrum* powder (mice model) [118]	- Mood and cognitive modulation in healthy adults after intake of *R. nigrum* juice or powder. Effect linked to the inhibition of platelet monoamine oxidase by sarmentosin and derivatives found in black currants [113] - Antioxidant and antiatherogenic effects after 2 h or 1-wk daily intake of *R. nigrum* drink by healthy adults [119]
American cranberry (*Vaccinium macrocarpon*)	- Phenolic compounds (anthocyanins, flavonols, flavanols, proanthocyanidins, phenolic acids) [104] - Triterpenoid compounds (ursolic, oleanolic, betulinic acids), phytosterols (β-sitosterol, stigmasterol) [104]	- Antimicrobial effect of *V. macrocarpon* phenolic compounds by inhibiting *Escherichia coli* adhesion [107] - Moderate antimicrobial effect against gram-positive and gram-negative oral pathogenic bacteria of fruit juice extracts [120] - Antioxidant activity correlated with the anthocyanins, flavonols, and triterpenoids contents [121]	- Improvement of the antioxidant status after intake of *V. macrocarpon* extract (ethylene oxide-treated rat model) [122] - Hepatoprotective and anti-inflammatory effects of *V. macrocarpon* phytochemical extract (rat model) [123] - Chemopreventive effect of phenolic and triterpenoid-sterols rich extracts from *V. macrocarpon* (colon cancer mice model) [124]	- Reduction of urinary tract infection (UTI) after daily intake of proanthocyanidin-rich *V. macrocarpon* extract (capsule) by women with recurrent UTI for 1-y period [125] - Gut microbiota modulation (high bifidogenic effect) after intake of high-polyphenol and oligosaccharides cranberry extract in healthy individuals (daily intake for 4 d) [126] - Improvement of cardiometabolic risk factors and hepatic steatosis in patients with nonalcoholic fatty liver (daily intake for 6 mo of cranberry powder supplement) [127]

Unless indicated, all information corresponds to the edible food fraction.

Abbreviation: ACE-I, angiotensin I-converting enzyme.

Furthermore, polar compound-rich extracts from chokecherry, currants, and cranberry exhibited anti-inflammatory, antihyperglycemic, antihypertensive, and antimicrobial activities, respectively [50,115,120]. Nonpolar compounds such as tocopherols, di and triterpenoids, phytosterols, and carotenoids have also been detected in native berries [46,50,51,104] (Table 3). Chokecherry and cranberry nonpolar fractions have been linked to the in vitro improvement of diabetic microvascular complications and in vivo chemopreventive effects, respectively [50,124]. In addition, chokecherry barks and currant leaves present antimicrobial and antioxidant potential [112,114]. Some studies in humans have reported the cognitive modulation potential of *R. nigrum* juice or powder [113], and the antimicrobial, gut microbiome modulation, and reduction of cardiometabolic risk factors of cranberry supplements [[125], [126], [127]].

As shown in TABLE 2, TABLE 3, in vivo and clinical studies are still very limited, especially in the case of native maize, wild rice, saskatoon, and chokecherry. Nevertheless, the scientific research is increasingly validating the indigenous knowledge about the traditional use and consumption of North American indigenous foods.

## Nutritional Composition and Health-Relevant Functionality of Some Andean Indigenous Grains

### Nutritional composition

The Andean region is distinguished by the presence of great food diversity including native grains which have been considered staple foods since pre-Hispanic times. The nutritional composition at macronutrient and micronutrient levels of some Andean grains is shown in Table 4.

**TABLE 4:** Nutritional composition of Andean indigenous grains (in 100 g of whole raw edible portion, dry weight, DW)

Compound	Unit	Andean Maize (*Zea mays* L.)	*Tarwi* (*Lupinus mutabilis* Sweet)	Quinoa (*Chenopodium quinoa*)	Andean Amaranth (*Amaranthus caudatus* L.)	Cañiwa (*Chenopodium pallidicaule* Aellen)
Origin		“Chullpi” race Peru	Peru	Red variety Pasankalla Peru	Cupi variety Peru	Centenario variety Peru
Macronutrients						
Protein	g	9.6	45.4	14.7	17.9	13.2
Total lipids	g	5.3	17.9	7.2	8.9	8.5
Total carbohydrates	g	68.4^1^	32.7	65.6^1^	54.3^1^	69.7^1^
Total dietary fiber	g	14.8	NR	9.9	14.0	6.5
Ash	g	1.9	4.0	2.6	5.0	2.0
Micronutrients						
Calcium, Ca	mg	1.0	NR	76.3	33	31
Iron, Fe	mg	1.9	7.0	3.3	5.5	5.6
Magnesium, Mg	mg	94	NR	NR	NR	NR
Phosphorus, P	mg	NR	NR	NR	NR	NR
Potassium, K	mg	NR	NR	NR	NR	NR
Sodium, Na	mg	NR	NR	NR	NR	NR
Zinc, Zn	mg	1.6	4.9	3.3	2.4	1.4
Copper, Cu	mg	0.1	0.8	NR	NR	NR
Vitamin C, total ascorbic acid	mg	NR	NR	NR	NR	NR
Complex B^2^	mg	NR	NR	NR	NR	NR
Vitamin A, RAE	μg	NR	NR	NR	NR	NR
Vitamin E (α-tocopherol)	mg	NR	NR	NR	NR	NR
References		[128]	[129,130]	[131]	[131]	[131]

Abbreviation: NR, not reported.

1 Only digestible carbohydrates.

2 Includes the sum of thiamin, riboflavin, niacin, pantothenic acid, Vit B-6-piridoxin, and total folate (B9).

Andean maize diversity is represented in different races. A race is defined as a genetically diverse material adapted to specific geographical region [132]. Andean maize races have shown one of the highest phenotypic diversity in the world, represented by a myriad of kernel pigmentations, ear and kernel morphologies, sizes, and traditional cultural uses [133]. The “Kculli” race (purple pigmented kernel and cobs), for example, is used for the preparation of nonfermented or fermented beverages named “chicha,” whereas the “Chullpi” race (yellow-pigmented kernel) is roasted and consumed as snacks or with other traditional food dishes [134].

Maize is a source of carbohydrates, protein, dietary fiber, and lipids [128] (Table 4). Andean maize shows higher dietary fiber (14.8% DW), but lower mineral contents than values reported for yellow maize (Table 1). This reveals differences due to the origin, genetic factors, among others, that should be studied case by case. Differences in the macronutrient and mineral concentrations have been reported even among Peruvian Andean maize races with variable kernel pigmentations [128]. In addition, Andean maize has shown better essential amino acid profiles in terms of leucine and tryptophan concentrations than commercial maize [128].

*Lupinus mutabilis* Sweet, also known as “tarwi,” “chocho,” or Andean lupin, is a legume that belongs to the *Fabaceae* family. It is the only American lupin species native from the Andean region, and is traditionally cultivated in Peru, Ecuador, and Bolivia since ancestral times [135]. *L. mutabilis* has been first domesticated in the Peruvian northern area [136] and is adapted to grow from 2000 to 3800 m [137]. Andean “tarwi” grains contain remarkable protein and lipid contents (45.4% and 17.9% DW, respectively) [129], which are comparable to soybean (*Glycine max*) seeds (39.9% and 21.8% DW for total protein and lipids, respectively), and are even higher than those found in other European *Lupinus* species such as *L. luteus*, *L. albus*, and *L. angustifolious* [41,138]. In addition, “tarwi” is considered a naturally biofortified crop due to its adequate levels of Fe, Zn, and Cu [130]. Iron contents (7 mg/100 DW, Table 3) are comparable to those observed in beans and pumpkin (9.3–10 mg/100 DW, Table 1).

Quinoa (*Chenopodium quinoa* Willd.), *cañiwa* (*Chenopodium pallidicaule* Aellen), and amaranth (*Amaranthus caudatus* L.) belong to the *Amaranthaceae* family and are known as pseudocereals which are dicotyledonous species with physical similarity to cereals (monocotyledonous grains). In the case of quinoa, Peru, Bolivia, and Ecuador are the countries with the highest production quantity worldwide, although this crop is also cultivated in lower quantities in non-Andean countries such as Buthan, reflecting its adaptation to other geographical regions [139]. Pseudocereals have shown better nutrient balance compared with cereal grains, particularly higher protein contents and adequate amino acid profile quality [140,141]. Protein levels in quinoa and amaranth vary from 14.7% to 17.9% DW [131], which are higher than those found in maize (9.6% DW, Table 4) and wheat (13.3% DW) [142]. All essential amino acids are present in quinoa and amaranth, showing higher contents of cereal and legume-limiting amino acids such as lysine and methionine than wheat and rice grains [140]. Depending on the variety, lower or not detectable levels of celiac-related prolamins have been found in Andean pseudocereals, increasing the interest on their use for different food applications [141].

In general, the information about the nutritional composition of Andean grain diversity, especially concerning mineral and vitamin profiles, is very scarce or not available (Table 4). This is very critical considering the high incidence of micronutrient deficiency among Andean populations as previously mentioned.

### Health-relevant functionality

The scientific evidence about the bioactive profiles and functionalities relevant for the health of some Andean indigenous grains is presented in Table 5. Main bioactive compounds identified in Andean maize correspond to polyphenols, carotenoids, and phytosterols [[143], [144], [145]]. Among phenolic compounds, anthocyanins are found at higher concentrations in purple maize types (“Kculli” race). In contrast to other blue/red maize varieties as those from North American regions, anthocyanins in Andean purple maize are not only accumulated in kernel pericarps but also in cobs. The total anthocyanin content determined by liquid chromatography was around 15 times higher in the cob than in kernels from Andean purple maize grown at 2800 m (64.4 and 983.0 mg/100 g DW expressed as cyanidin chloride in kernel and cob samples, respectively) [146]. Andean purple maize generally has the highest anthocyanin contents among maize from other worldwide regions [147]. Major anthocyanins in Andean purple maize are cyanidin-3-glucoside, pelargonidin-3-glucoside, peonidin-3-glucoside, their acylated derivatives along with other flavanol-condensed anthocyanin types which are concentrated in the pericarp [145]. On the basis of this potential, purple-pigmented Andean maize germplasm has been used for creating new high-anthocyanin maize varieties [148]. Accordingly, several studies aimed at evaluating the health benefits of Andean maize have focused mostly on the purple maize class (Table 5). Extracts of Andean purple maize have shown in vitro antiobesity and antioxidant effects [149,150]. Moreover, anthocyanin-rich purple maize extracts showed in vivo CVD protection and antihyperglycemic activity [151,152]. Only 1 clinical trial demonstrated the antihypertensive properties of the intake of a Peruvian purple corn extract [153]. Unlike the interest in the research of purple maize, little is known about the bioactive composition and health properties of the vast diversity of nonpurple Andean maize.

**TABLE 5:** Bioactive compounds and reported health-relevant properties associated to some Andean indigenous grains (studies from Andean foods)

Food	Bioactive compounds	Reported bioactivity		
		In vitro	In vivo	Studies in humans
Andean Maize (*Zea mays* L.)	- Phenolic compounds: flavonoids (anthocyanins, flavones, flavonols, flavonol-condensed anthocyanins), hydroxybenzoic acids, hydroxycinnamic acids [[143], [144], [145]] - Carotenoids: zeaxanthin, lutein, isomers of β-cryptoxanthin, violaxanthin, and neoxanthin [143] - Phytosterols (campesterol, β-sitosterol) [143]	- Antioxidant and antihyperglycemic (α-glucosidase inhibition) effects of Andean maize hydrophilic extracts correlated with the phenolic fraction [143] - Antiobesity (lipase inhibition) effect of a purple maize extract, and correlated with the anthocyanin contents [149] - Antioxidant effect in isolated mouse organs by a purple corn extract (decrease of lipid peroxidation and increase of the endogenous antioxidant enzymes) [150]	- Protection against cardiovascular disease and hepatic steatosis after the intake of diet enriched with purple corn extract (broiler model) [151] - Antihyperglycemic and pancreatic beta cell-protection activities of purple corn anthocyanins (mice model) [152]	- Antihypertensive effect (adults with mild-to-moderate hypertension) after daily intake of purple corn extract (capsule with 300 mg anthocyanins) for 3 wk [153]
Andean lupin *Tarwi* (*Lupinus mutabilis* Sweet)	- Phenolic compounds: isoflavones (genistein and its derivatives, mutabilein: 3'-methoxy-5,7-dihydroxyisoflavone); flavones (apigenin and luteolin derivatives); catechins; hydroxycinnamic acid derivatives [[154], [155], [156]] - Phenyletanoids (tyrosol and derivatives) [155] - Carotenoids (lutein, zeaxanthin); tocopherols (α, β, γ, δ) [157] - Proteins (γ-conglutin) and bioactive peptides [158,159] - Quinolizidine alkaloids (lupanine, sparteine and their derivatives) [138]	- Antioxidant response and anti-inflammatory stimulation of tarwi γ-conglutin in human monocyte cell cultures infected with *Leishmania* (Viannia) *peruviana* [158] - Antioxidant, antihypertensive (ACE-I inhibition), and antihyperglycemic (DPP-IV inhibition) effects of *tarwi* synthesized peptides, and peptidic fractions derived from protein hydrolysate [159,160] - Antimicrobial effect of “tarwi” hydroethanolic extract against urinary tract infections (uroepithelial cell model) by preventing cell adhesion and decreasing biofilm formation [161]	- Antidiabetic effect after oral intake of *tarwi* hydroethanolic extract by stimulating insulin release (diabetic rat model) (alkaloids may be involved) [162]	- Antihypertensive effect and HDL cholesterol increase in patients with type 2 diabetes with regular hypoglycemic oral treatment for 14 wk, followed by daily intake of “tarwi”-snack for an additional 14-wk period [163] - Antihyperglycemic adjuvant effect after 6-mo intake of “tarwi”-based snack in patients with diabetes with metformin treatment (decrease in glycated hemoglobin and BMI) [164] - Hypoglycemic effect after 90 min intake of debittered cooked “tarwi” or a purified alkaloid fraction (γ-conglutin or the alkaloid fraction may be involved) [165]
Quinoa (*Chenopodium quinoa* Willd.)	- Phenolic compounds: flavonoids (quercetin, kaempferol and their derivatives, isoflavones); phenolic acids [166,167] - Triterpenoid saponins, betacyanins (betanin, isobetanin), and phytosterols [[166], [168]] - Tocopherols (α, γ, δ) and carotenoids (lutein, zeaxanthin) [169,170] - Bioactive peptides [171]	- Antioxidant activity correlated with the flavonoid and total phenolic contents [172,173] - Antihyperglycemic effect (α-glucosidase inhibition) of the bound phenolic fraction (rich in ferulic acid and derivative, and quercetin) [174] - Antihyperglycemic (α-glucosidase and DPP-IV inhibition) and antihypertensive activities (ACE-I inhibition) of quinoa peptides and protein hydrolysates [[175], [176], [177]]	- Colon cancer mitigation after intake of quinoa protein or its hydrolysate by partially regulating gut microbiota (mice model) [178] - Antihyperglycemic effect of the bound phenolic fraction (50 mg/kg) applied by oral gavage (mice model). Comparable decrease of postprandial blood glucose with acarbose (20 mg/kg) [174] - Antioxidant, anti-inflammatory effects, and protection of liver and kidney damage due to insulin resistance after intake of quinoa-based diet (rat model) [179]	- Improvement of hyperglycemia and lipid metabolism in patients with impaired glucose tolerance after daily intake of a quinoa-based diet for 1 y [180]
Andean Amaranth (*Amaranthus caudatus* L.)	- Phenolic compounds (flavonols, phenolic acids and derivatives) [181] - Betacyanins (amaranthine, iso-amaranthine) [182] - Triterpenoid saponins [183] - Bioactive peptides [176,184] - Phenolic compounds (phenolic acids and flavonols) and betacyanins in stalks, leaves, flowers, and sprouts of *A. caudatus* [185]	- Antioxidant and high digestibility of *A. caudatus* protein hydrolysates [186] - Antioxidant and anticancer activities (breast cancer cells) of peptides derived from *A. caudatus* protein hydrolysates [184]. Anticancer effect of *A. caudatus* lectin fraction (osteosarcoma cell model) [187] - Antihypertensive (ACE-I inhibition) and antioxidant activities of *A. caudatus* protein hydrolysates [176]	- Antihyperglycemic effect after intake of *A. caudatus* extract (rich in sugars, traces of phenolic compounds and amino acids) by improving insulin secretion (diabetic rat model) [188]	- Low glycemic index (48.63–52.19) after 2 h consumption of amaranth-based snack bar (with 90% of fermented *A. caudatus* flour) by healthy and diabetic adults, respectively [189,190]
*Cañiwa* (*Chenopodium pallidicaule* Aellen)	- Phenolic compounds (flavonols, flavanols, phenolic acids) [182,191] - Phytosterols (stigmasterol, stigmastedienol acetate, β-sitosterol acetate) [192] - Triterpenoid saponins [193] - Bioactive peptides [194]	- Protein showed high digestibility after simulated in vitro digestion, and its hydrolysates increased cellular antioxidant activity in human hepatocellular carcinoma and colorectal adenocarcinoma cells [195] - Antioxidant and antihyperglycemic (α-glucosidase and α-amylase inhibition) of a hydroethanolic extract [196] - Antimicrobial activity against *E. coli*, *S. aureus*, and *C. albicans* by *C. pallidicaule* peptides [197] - Antioxidant and antihypertensive (ACE-I inhibition) activities of *C. pallidicaule* protein hydrolysates and peptides [194]	- Neuroprotective effect after application of *C. pallidicaule* flour water suspension via orogastric (mice model). Reduction of ethanol-derived histological damage in brain and cerebellum [198]	- Not found

Unless indicated, all information corresponds to the edible food fraction.

Abbreviations: ACE-I, angiotensin I-converting enzyme; DPP-IV, dipeptidyl peptidase IV enzyme.

Major phenolic bioactive compounds detected in “tarwi” are flavonoids (Table 5). From the 21 flavonoids identified in Peruvian samples by liquid chromatography coupled to MS, 13 corresponded to isoflavones (genistein derivatives), and 8 were flavones (apigenin and luteolin derivatives) [154]. Genistein and mutabilein (3′-methoxy-5,7-dihydroxyisoflavone) isoflavones along with their derivatives have been also found in grains grown at greenhouse conditions and derived from 2 Colombian “tarwi” ecotypes [156]. Additionally, phenyletanoids, carotenoids, and all chemical forms of tocopherols have been reported in Andean lupin [155,157]. “Tarwi” protein fractions and derived bioactive peptides have also played a role in several biological activities (Table 5). The γ-conglutin fraction was related to in vitro antioxidant and anti-inflammatory effects in response to *Leishmania peruviana* infection [158]. Synthesized peptides and peptidic fractions from “tarwi” protein hydrolysates have exhibited antioxidant, antihypertensive, and antihyperglycemic potential in vitro [159,160]. The consumption of “tarwi”-based snacks has improved different metabolic biomarkers, complementing the regular treatment of individuals with diabetes [163,164]. These effects have likely been attributed to “tarwi” protein and dietary fiber, although the exact mechanisms are still unknown [163].

Quinolizidine alkaloids (QA) are L-lysine-derived secondary metabolites which confer a bitter taste in “tarwi” grains [199]. There is evidence of acute and chronic toxicity when QA are consumed above certain doses [138,199]. Thus, grains are commonly washed several times to eliminate these compounds before consumption. Nevertheless, some in vivo and human studies are associating the alkaloid fraction (in non-toxic concentrations) with the modulation of hyperglycemia [162,165] (Table 5). In fact, QAs such as sparteine and lupanine have also shown other pharmacological properties such as antirhythmic, anticonvulsionant, antimicrobial activities, and antidiabetic effects [[200], [201], [202], [203]]. This potential requires major research considering specific QA concentrations and profiles.

The scientific advances about the health-related properties of Andean pseudocereals have been more focused on quinoa than in amaranth and “cañiwa” (Table 5). Several phenolic compounds, mainly flavonoids such as quercetin, and kaempferol glycosides, along with isoflavones have been detected in quinoa grains [166,167]. Among lipophilic compounds, tocopherols, carotenoids, and phytosterols have been identified in quinoa [168,169]. Other bioactive compounds such as saponins, betacyanins, and bioactive peptides have also been reported in quinoa grains (Table 5). Amaranth and “cañiwa” are also sources of several biologically active compounds including polyphenols, saponins, phytosterols, and bioactive peptides (Table 5). The betacyanins amaranthine and isoamaranthine have been reported in Peruvian amaranth samples [182]. Interestingly, other edible parts (stalks, leaves, flowers) of the Andean amaranth plant are also sources of phenolic compounds and betacyanins [185]. Traditionally, Andean communities have used quinoa and amaranth leaves, stalks, and grains not only for nutrition but also for medicinal purposes. Quinoa stalks and leaves have been used as a purgative, for colic relief, as anti-inflammatory agents, and as analgesics [204,205].

Due to the higher protein contents in Andean pseudocereals compared with common cereals, their protein fractions, and derived peptides have been related with several biological activities. Quinoa peptides or protein hydrolysates showed in vitro antihyperglycemic and antihypertension properties, and in vivo anticancer and gut microbiome modulation [175,177,178]. In addition, amaranth protein hydrolysates and a lectin fraction have shown in vitro antioxidant and anticancer activities, respectively [[186], [187]]. “Cañiwa” peptides exhibited antimicrobial properties, whereas protein hydrolysates promoted the cellular antioxidant activity in cancer cells [195,197].

Other bioactive compounds such as the quinoa phenolic fraction (bound phenolic compounds) have also displayed potential for hyperglycemia treatment according to in vitro and in vivo results [174]. The intake of diets based on whole quinoa and amaranth grains has shown promising results for the prevention or improvement of diabetes [180,189]. Nevertheless, in vivo and clinical studies are still scarce as discussed previously for the other indigenous foods examples.

## Revitalization of North American and Andean Traditional Food Systems for Health Improvement

The information presented in previous sections about some North American and Andean indigenous plant-based foods strongly supports their excellent and high-value nutritional and health-relevant potential. The scientific research is increasingly validating ancient indigenous knowledge linked to the cultivation and consumption of culturally relevant foods. However, given the great diversity of indigenous foods in both regions of the Americas, these scientific advances are still very limited, or the information simply does not exist. It has been reported that only 18.4% of indigenous North American food plants have some contemporary scientific information about their nutritional value [206]. The macronutrient and micronutrient composition, especially related to the mineral and vitamins contents of Andean grains, is scarce as evidenced in the current paper. The same limitations occur in the case of research focused on the validation of health properties of indigenous foods based on in vivo and clinical trials.

Thus, the scientific research is critical for continuing the advance on the characterization of the indigenous food diversity per region. The lack of information or interest on the value of indigenous foods, along with the replacement of traditional diets by highly processed foods, among other factors as previously stated, is compromising the preservation of indigenous food diversity and associated traditional food systems [[26], [27], [28], [29], [30], [31], [32], [33]].

The revitalization of both North American and Andean food systems requires a multidisciplinary focus, integrating the participation of multiple stakeholders at both geographical regions. Actions aimed at this goal should primarily consider the participation of indigenous communities, including their culture and knowledge. Some general key points targeted to revitalize indigenous food systems are given in the following paragraphs, but each situation should be evaluated considering its particular and unique contexts.

### To promote the conservation of indigenous food diversity

Most indigenous North American and Andean foods are currently considered neglected or underutilized in their own centers of origin [206,207]. This is leading to a high risk of genetic erosion, that is impairing food security and indigenous knowledge associated with food production, preparation, and cultural uses. The knowledge on how to use many indigenous American wild edible plant species has been lost [206]. Only 13.5% of indigenous inhabitants from some southern Andean provinces in Peru remember the ancestral traditional knowledge about medicinal uses of quinoa [204]. The preservation of agrobiodiversity is the foundation for improving sustainable agricultural systems and resilience [208], which in turn would positively impact indigenous health and well-being. Therefore, the multidisciplinary scientific research should be associated with initiatives for preserving food diversity and involving indigenous communities.

In the case of an Andean country such a Peru, a list of prioritized highly diverse crops, including some Andean grains, has been identified [209]. Concurrently, a scheme named payment for agrobiodiversity conservation services (PACS) has been applied to some prioritized crops such as quinoa for in-situ on-farm conservation [210]. This strategy was aimed at rewarding Andean communities for cultivating and preserving indigenous food diversity linked to their traditional food systems [210]. In addition, this mechanism allows a high level of participation of indigenous farmers by defining their own conditions and targeting a fair benefit sharing [211]. Moreover, in-situ and ex situ integrated conservation platforms involving “indigenous custodian farmers” have been suggested to be linked to PACS for more effective and sustainable results [212].

Some broader implemented initiatives in Canada such as the “Coastal Guardian Watchmen”, and “Indigenous Guardians Program” are targeting to preserve food diversity and uses, their traditional practices, to protect their ecosystems, and to reconnect involved indigenous American people with their lands and environments [8]. In the United States, the project called “Iroquois White Corn Project” has the goal of restoring traditional white corn (Tuscarora white) cultivation, intake, and distribution among American Indian groups [213]. In another initiative, a network of “Indigenous Corn Keepers” is preserving a collection of thousands of native corn varieties within the Onondaga Nation Farm [214]. The objective is not only to guarantee food security among indigenous communities, but also to reconnect people with their sacred seeds and related cultural traditions [214].

### To encourage the consumption of indigenous foods and food design based on indigenous foods

Along with the initiatives to preserve indigenous foods, efforts should also be targeted to revive the traditional indigenous food preparations, or to propose the design of healthy innovative foods based on the diversity of indigenous foods. The rescuing and documentation of food preparations based on indigenous foods seem to be an important strategy. Several historical preparations from indigenous foods derived from the Three Sisters system have been reported [38]. Authors suggested that indigenous foods and traditional preparations may be the base for the design of healthier diets and innovative gastronomy [38]. Furthermore, a recipe book entitled *A Harvest of Recipes with USDA Foods* has been published by the USDA Food and Nutrition Service, Food Distribution Division, and was developed for Food Distribution Program on Indian Reservations participants and staff [215]. This book compiles healthy traditional recipes adapted from regional indigenous recipes and includes the nutrition facts for each preparation [215]. In Andean countries, similar initiatives also exist targeting low-cost, nutritious, and healthy preparations based on culturally acceptable indigenous foods [216]. The cultural importance and ancient culinary preparations based on quinoa in Bolivia have also been reported [217].

The food development considering modern preferences and based on indigenous foods is another alternative for promoting their intake. Pemmican made with dried meat (bison, moose, among others), meat or fish fat, and different dried berries such as saskatoon berries, chokeberries, or cranberries, is an innovative indigenous food designed to face extreme environments and work activities [218]. This food has been translated into diverse, healthier, meat-based, highly nutritious, and high antioxidant snacks [219]. Along with berries, other plant based–indigenous foods could be incorporated. Similarly, different sausage formulations based on low-fat and cholesterol lama (*Lama glama* L.) meat, pecans (*Carya illinoinensis*), and cañiwa (*Chenopodium pallidicaule*) have been proposed for the Andean market [220].

Different food formulations prepared with Andean grains have also been reported and may play a role on the health of indigenous communities and local consumers. Some examples are: baby purees elaborated with pumpkin, quinoa, amaranth, and potato flours [221]; gluten-free maize extrudates enriched with “tarwi” flour and pecan nut with improved contents of phenolic compounds [222]; gluten-free pasta made with “tarwi” and rice flours [223]; bread alternatives enriched with amaranth and “cañiwa” [224]; and functional beverages with milk, “tarwi,” and oatmeal [225]. Because all these products are nutritionally balanced and may have promising health effects, further in vivo and clinical trials are needed to prove these properties.

## Conclusions

Domesticated plants have provided food and medicines to communities across the world and have been integral part of human ecology since the dawn of organized societies. This conference paper focused on examples of plant-based foods that have been used among indigenous North American and Andean societies covering Americas. Various local and wider societal challenges of contemporary times have caused a decrease in their consumption, leading to several diet-related health challenges such as NCDs and associated nutritional deficiencies. The challenges associated with these health and ecosystems burdens such as climate change are also compromising the preservation of indigenous food diversity which also includes all the indigenous concepts associated with traditional food systems and food sovereignty. This paper therefore focused and summarized the scientific advances associated with the nutritional and health-relevant potential of some indigenous foods from these regions such as the Three Sisters crops, wild rice, and some native berries, and diverse Andean grains. Furthermore, some initiatives aimed at preserving indigenous food diversity and associated food systems in each region have also been discussed. The scientific evidence presented here validates the indigenous knowledge from both regions linked to the consumption of native foods for maintaining good nutrition and health, which facilitates the indigenous communities to reconnect with their culture and traditions. Building from these insights, more scientific research and efforts are needed from multiple stakeholders associated closely with the indigenous communities to continue the revitalization of indigenous food systems and their health benefits.

## Author contributions

LGR, conceptualization, writing original draft, review and editing; KS, conceptualization, review and editing; MM, conceptualization, review and editing. All authors read and approved the final manuscript.

## Funding

The authors reported no funding received for this study.

## Conflict of interest

The authors report no conflicts of interest.
